# Occupational exposure to physicians working with a Zero-Gravity™ protection system in haemodynamic and electrophysiology labs and the assessment of its performance against a standard ceiling suspended shield

**DOI:** 10.1007/s00411-022-00968-4

**Published:** 2022-02-26

**Authors:** Joanna Domienik-Andrzejewska, Mateusz Mirowski, Marek Jastrzębski, Tomasz Górnik, Konrad Masiarek, Izabela Warchoł, Włodzimierz Grabowicz

**Affiliations:** 1 Radiation Protection Department, Nofer Institute of Occupational Medicine in Lodz, Łódź, Poland; 2 Medical College, 1St Department of Cardiology, Jagiellonian University, Interventional Electrocardiology and Hypertension, Kraków, Poland; 3 Department of Invasive Cardiology and Cardiodiabetology, Medical University of Lodz, Łódź, Poland

**Keywords:** Dosimetry, Radiation protection, Zero-Gravity™ radiation protective system, Interventional cardiology, Radioprotective performance, Ceiling suspended lead shield

## Abstract

A two centre clinical study was performed to analyse exposure levels of cardiac physicians performing electrophysiology and haemodynamic procedures with the use of state of the art Zero-Gravity™ radiation protective system (ZG). The effectiveness of ZG was compared against the commonly used ceiling suspended lead shield (CSS) in a haemodynamic lab. The operator’s exposure was assessed using thermoluminescent dosimeters (TLDs) during both ablation (radiofrequency ablation (RFA) and cryoablation (CRYA)) and angiography and angioplasty procedures (CA/PCI). The dosimeters were placed in multiple body regions: near the left eye, on the left side of the neck, waist and chest, on both hands and ankles during each measurement performed with the use of ZG. In total 29 measurements were performed during 105 procedures. To compare the effectiveness of ZG against CSS an extra 80 measurements were performed with the standard lead apron, thyroid collar and ceiling suspended lead shield during CA/PCI procedures. For ZG, the upper values for the average eye lens and whole body doses per procedure were 4 µSv and 16 µSv for the left eye lens in electrophysiology lab (with additionally used CSS) and haemodynamic lab (without CSS), respectively, and about 10 µSv for the remaining body parts (neck, chest and waist) in both labs. The skin doses to hands and ankles non-protected by the ZG were 5 µSv for the most exposed left finger and left ankle in electrophysiology lab, while in haemodynamic lab 150 µSv and 17 µSv, respectively. The ZG performance was 3 times (*p* < 0.05) and at least 15 times (*p* < 0.05) higher for the eye lenses and thoracic region, respectively, compared to CSS (with dosimeters on the apron/collar). However, when only ZG was used slightly higher normalised doses were observed for the left finger compared to CSS (5.88*e *− 2 Sv/Gym^2^ vs. 4.31* e *− 2 Sv/Gym^2^, *p* = 0.016). The study results indicate that ZG performance is superior to CSS. It can be simultaneously used with the ceiling suspended lead shield to ensure the protection to the hands as long as this is not obstructive for the work.

## Introduction

Due to both high doses from single procedure and a considerable workload, interventional cardiology practices involve relatively high doses for physicians performing the procedures (Vaño 2003, 2006; Kim and Miller 2009; Martin 2009; Domienik-Andrzejewska et al. 2018). For this reason, there is fast technological development of methods and tools helping in reducing doses to the medical staff (Mirowski 2021). The latest innovations include lead-free aprons and state of the art shields design to protect the prime operator, such as radiation protective cabin or Zero-Gravity system (ZG). The two latter ones reduce exposure of the operator while additionally eliminating orthopaedic injuries (Dragusin et al. 2007; Savage et al. 2013; Haussen et al. 2016; Maleux et al. 2016). Therefore, these systems are also a good solution for the problem of physicians’ chronic back pain due to working in a standard lead apron. While the radiation protective cabin is mainly dedicated for radiofrequency ablations (RFA) procedures (due to limited access to the catheter) the ZG system can be successfully used during all fluoroscopy guided procedures. In the ZG system, the ceiling suspended gantry of the system eliminates the weight and allows movements in all directions. Thanks to the side and front lead glass (0.5 mm Pb), ZG should provide optimal protection to the eye lens, head and neck region. Moreover, the lead apron protects physician from the collar up to the tibia with twice the thickness of the standard lead apron. The ZG system can be used simultaneously with ceiling suspended lead glass providing potentially even more enhanced protection for the primary operator. The aim of the paper was to determine the typical exposure levels of physician’s working with a ZG system during electrophysiology procedures [such as radiofrequency ablations (RFAs) or cryoablations (CRYAs)] and haemodynamic procedures [such as angiography (CA) and angioplasty (PCI)] and to compare the protective effectiveness of this system with standard solutions such as the ceiling suspended lead shield used in most interventional rooms.

## Materials and methods

The study was performed in two large medical centres in Poland. In the first centre the ZG system is permanently installed in a electrophysiology lab and it is the only centre in Poland to own this system. In the second centre, the system was installed in a haemodynamic lab for a trial period of 2 months. In the electrophysiology lab the new system was used simultaneously with the ceiling suspended lead glass (0.5 mm Pb) and the table curtain (0.5 mm Pb), while in the haemodynamic room, originally equipped with the same standard radiation covers, the ceiling suspended lead glass was disassembled and its suspension system was used to mount the ZG system for the purpose of this study.

One of the two physicians performing haemodynamic CA and PCI procedures and involved in the measurements performed about 88% of procedures using the new system, while out of the four physicians in the electrophysiology lab, the most experienced, performed 42% of procedures. In total, 29 measurements were performed with the ZG system during randomly selected procedures; among them 15 in haemodynamic lab and 14 in electrophysiology one. To detect exposure from low dose procedures, for each measurement the doses from several procedures were cumulated (minimum two and maximum six). Therefore, the measurements performed include in total doses from 105 procedures (36 RFA or CRYA and 69 CA and PCI). The position of the main operator in haemodynamic and electrophysiology lab during each procedure corresponded to the position of the physician inserting the catheter into the radial artery (about 60 cm from the X-ray tube to the physician measured along the treatment table) and the femoral artery (80–85 cm depending on the patient height), respectively.

The exposure of every physician was monitored with eight dosimeters placed in various positions on his/her body during each individual measurement with the ZG system. Three whole body thermoluminescent dosimeters were attached to the left side of the neck, chest and waist. The dedicated eye lens dosimeter, mounted on a band was placed near the left eye closest to the radiation source. Additional four loose TL dosimeters were attached to each finger and ankle to evaluate the doses to extremities (a priori considered as not protected by the ZG system). The dosimeters were calibrated in standard reference beams (PN-ISO 4037-3:2004) N-80 or N-100 against individual dose equivalent Hp(3) and Hp(0.07) or Hp(10), respectively. The lower detection limit (LDL) for whole body dosimeter was 0.020 mSv, for the eye lens 0.010 mSv and 0.005 mSv for loose TL detectors on fingers and legs. All doses below appropriate LDL were replaced by the values of the latter.

The information on the radiation emitted by the X-ray unit and time of fluoroscopy for each single procedure was also collected in terms of the dose-area product (DAP), cumulative dose (CD) and fluoroscopy time (FT). Moreover, the weight and height of each patient were also collected to determine corresponding body mass index (BMI) for further evaluation of its influence on the obtained results.

For research purposes, the distributions of average Hp(10), Hp(3) and Hp(0.07) doses per procedure in multiple body regions and the corresponding doses normalised to DAP were analysed. Normalization to DAP applied in the study made it possible to exclude the dependence of the doses on both the duration of the procedure and the amount of radiation utilized during the examination.

Finally, the effectiveness of the ZG system was verified by comparison of the physicians’ exposure during haemodynamic procedures performed using ‘zero gravity’ to the exposure during procedures performed by the same group of physicians working in the standard lead apron and using the ceiling suspended lead shield. To this end the normalized doses measured on the chest above the lead apron, on eye lens and fingers were compared. The same strategy was planned for RFA/CRYA procedures; however, due to COVID-19 restrictions further measurements (with standard lead apron, thyroid collar and CSS) could not be performed, and therefore, it was impossible to analyze the shield efficiency in the radiation fields used during ablations. However, it is not expected to differ significantly from the one observed in the haemodynamic room.

To evaluate the ergonomic properties of the ZG shield, two questions were asked, specifying both the comfort of ‘wearing’ the new shield as well as its possible limitations from the medical or practical point of view as compared to the advantages vs. disadvantages of a standard lead apron.

## Statistical analysis

Differences between two types of doses (with the CSS and with the ZG system instead the CSS) were assessed using *t*-Student or Mann–Whitney *U* test depending on the character of data distribution (normal or non-normal). A *p* value < 0.05 was considered significant. Statistical analyses were performed using the R software (R-Core Team 2017).

## Results

More than 90% of measurements performed in the electrophysiology room were below the lower detection limit (LDL), while the respective value was 30% for the haemodynamic room. In particular, in the former lab, all doses measured on the neck, chest, waist and left eye lens area were below LDL in contrast to only few or none doses in the haemodynamic lab. In the remaining body regions (left and right fingers and ankles) in the case of several measurements the doses reached or were above the lower detection limit in the electrophysiology lab, while in the haemodynamic lab, all doses were well above the limit.

**Fig. 1:**
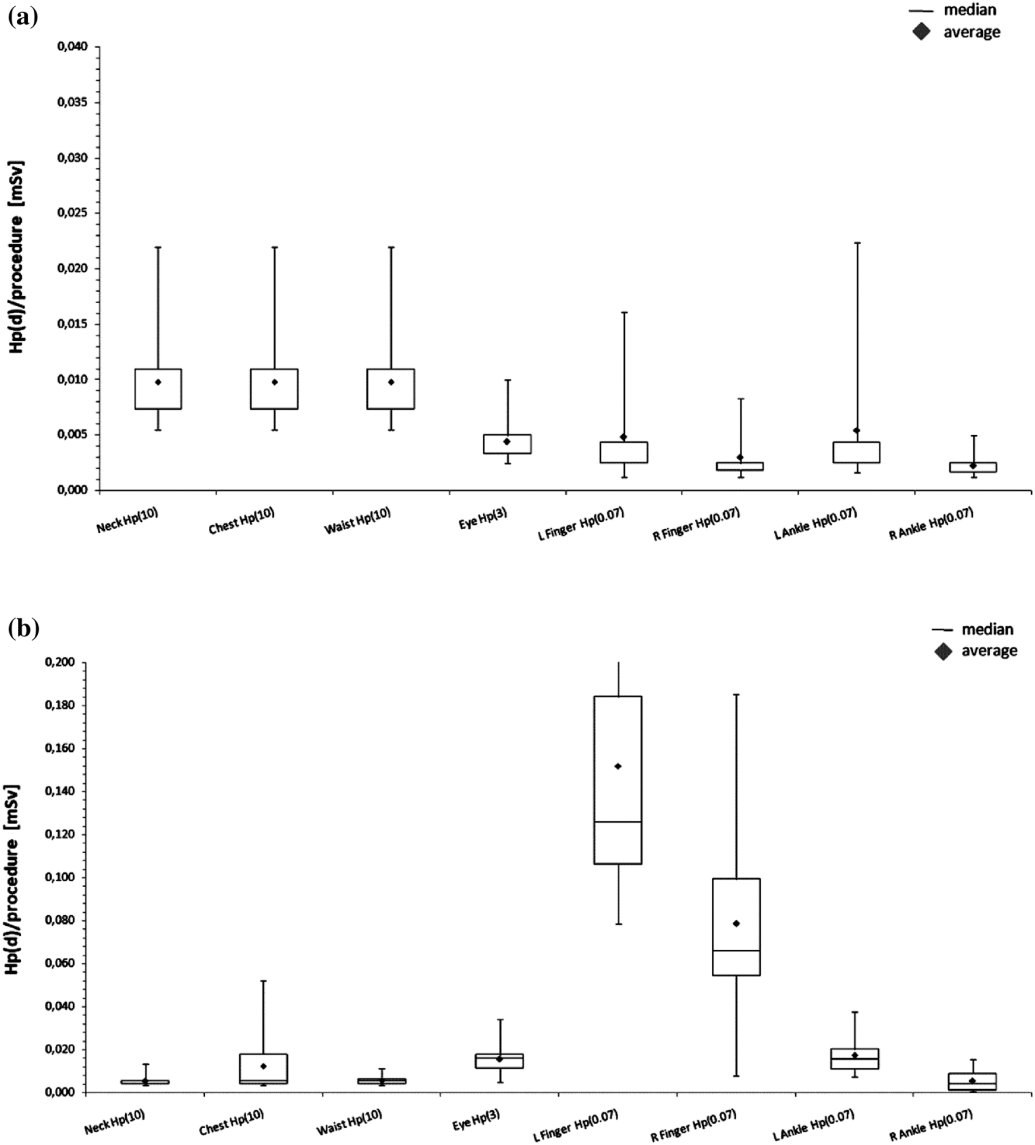
**a** Distribution of doses per procedure in various body regions: neck, chest, waist, left eye lens, both fingers and ankles in electrophysiology. **b** Distribution of doses per procedure in various body regions: neck, chest, waist, left eye lens, both fingers and ankles in haemodynamic lab

In Table 1, the values of average level of radiation emitted by the X-ray tube during a single procedure of given type (expressed in terms of DAP and CD) are presented as well as typical fluoroscopy times and BMI (Body Mass Index) of the patient—a parameter characterising the size of the patient. The distributions of doses per procedure to various body regions for both physicians working in electrophysiology and haemodynamic labs are presented in Fig. 1a, b, respectively. No statistically significant differences were found between the amount of radiation used or average BMI of the patient for CA/PCI procedures performed with CSS and with ZG system instead of CSS. The only difference between both procedure types (CSS vs. ZG) was found for the FT parameter which includes the exposure time acquired in the fluoroscopy mode but not in the cine mode and, consequently, does not take into account the exposure time for the whole procedure.

**Table 1:** Average amount of radiation emitted during single procedure of given type performed with ZG and with CSS, expressed in terms of DAP and CD, average typical fluoroscopy time (FT) per procedure and BMI of the patient

Procedure type	mean ± SD			BMI
	DAP [µGym^2^]	CD [mGy]	FT [min]	
CA/PCI with ZG	2706 ± 1878	545 ± 386	5.1 ± 4.4	28.2 ± 5.3
CA/PCI with CSS	3645 ± 3080	735 ± 632	8.0 ± 5.7	28.7 ± 4.0
Difference	*p* = 0.200	*p* = 0.210	***p* = 0.019**	p = 0.189
RFA/CRYA with ZG	1598 ± 944	n.a*	13.8 ± 6.2	29.4 ± 3.5

*SD* standard deviation, *n.a.* not available

**Fig. 2:**
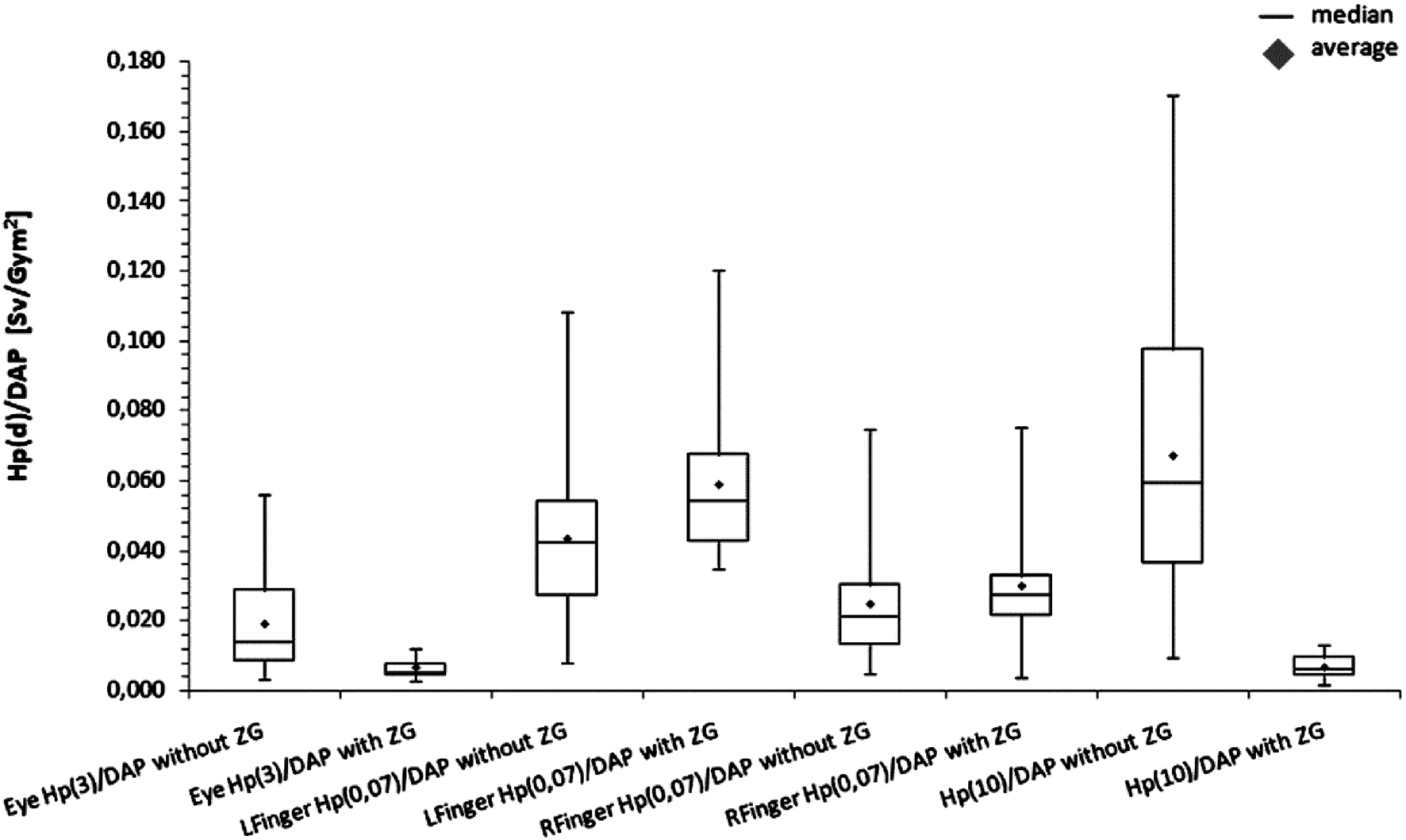
Comparison of doses normalised to DAP obtained with ZG and with CSS (without ZG) in various body regions during haemodynamic procedures

The assessed doses per procedure in the areas shielded by the ZG system were low: the average whole body doses per procedure for the thorax were equal or lower than about 10 µSv, while for the left eye lens, they were even lower, equal or less than 4 µSv for electrophysiology, or 16 µSv for haemodynamic procedure. As for the doses that physicians received on parts of the body not protected by a ZG shield, such as hands and ankles, the values in the haemodynamic laboratory were 150 µSv and 79 µSv for the left and right finger, respectively, and 17 µSv and 5 µSv for the left and right ankle, respectively. In the electrophysiology lab, the corresponding values were 5 µSv and 3 µSv for left and right finger and 5 µSv and 2 µSv for left and right ankle. A comparison of normalized doses obtained by physicians when using the ZG system and standard ceiling transparent lead glass are presented in Fig. 2. Statistically significant differences were observed for doses to the eye lens (*p* < 0.05), the left fingers (*p* = 0.016) and Hp(10) doses measured on the chest (*p* < 0.05). Due to the use of ZG system the doses measured on the eye lens and the chest were significantly reduced—at least 3 times and 15 times, respectively, while for the left finger, a slight increase was observed (1.3-fold increase).

A short questionnaire study showed that the comfort of working with the ZG system is much higher than with the lead apron, although using this shield may slightly extend the duration of the procedure at least in case of a physician inexperienced in working with the ZG system. The possible reason is a limited visibility of the pedal activating the exposure.

## Discussion

The analysis of doses measured during haemodynamic procedures performed with the ZG and CSS system allowed to compare the effectiveness of the state-of-the-art radiation protection shields and the standard one, such as ceiling suspended lead glass. The measurements exhibited high attenuation of doses in the areas, where dosimeters were shielded by the ZG system; in particular, in the region of chest and the eye lens where at least 15-fold and threefold reduction of doses, respectively, is predicted. The effectiveness of the ZG system in reducing the exposure to the eye lens found in this study is lower than that reported by Savage et al. (2013) (66% vs. 87% reduction for eye &head region) and Zanca et al. (2021) (66% vs. 79%/83% reduction for the left eye in CA/PCI procedures) while higher than that reported by Haussen et al. (2016) (66% vs. 50%). In quoted studies, except for the Zanca et al. (2021), the operators performed different non-cardiac interventional radiology procedures. The difference suggests that the performance of the ZG system might depend on dynamic factors related to clinical practice, such as the projection types used, the physician’s position with respect to the center of X-ray field, exposure parameters depending on patient size and the treatment area etc. which are, to some extent, characteristic for procedure type but may also differ for procedures of the same type. According to results of the measurements performed on the phantoms presented in the report Dabin et al. (2021) and published in the paper by Zanca et al. (2021) the effectiveness of ZG system on the level of the eye lens is high and only slightly depends on the projection type at least for analysed in this study projections (PA, LAO 30, LAO 90); the dose reduction for the left eye lens ranges from 84% for PA projection to 96% for LAO 90 and for the right one from 75 to 92%, respectively. These values were also consistent with the results of computational simulations (Dabin et al. 2021). The effectiveness of ZG system may also change with the position of physician as it is reported in the paper by Ray et al. (2013); for the use of ZG system compared to standard shields (CSS and table side shield) the following eye lens doses normalised to DAP were reported: 0.0062/0.0066/0.0026 µSv/Gycm^2^ vs. 0.8137/1.4631/1.3228 µSv/Gycm^2^ for femoral, side and head access, respectively. However, one has to keep in mind that the procedures performed using various accesses in this study were not of the same type, and therefore, the position of physician might not be the only reason for reported differences. In general, this fact makes the analysis of the influence of dynamic factors on the performance of ZG-system difficult—reasonable comparison between results obtained in various studies is possible provided the clinical practices to be compared differ by one dynamic factor only which is hardly possible. In the case of our study, for example, the differences between eye lens doses normalised to DAP for CA/PCI and RFA/CRYA (0.629 µSv/Gycm^2^ vs. 0.496 µSv/Gycm^2^) might be due to few dynamic factors such as the shielding (only ZG vs. ZG and CSS), the access (radial vs. femoral) and projections used [large variety of different types vs. restricted to few (mainly PA)]. Their relatively high values with respect to other studies, 0.017 µSv/Gycm^2^ in Savage et al (2013), 0.006 µSv/Gycm^2^ in both Kwarcinski et al. 2020 and Ray et al. 2013 and 0.123 µSv/Gycm^2^ in Haussen et al. 2016, might be also due to relatively low sensitivity of TLDs and estimated LDL used to assess the exposure for RFA/CRYA (all measured eye lens doses were below LDL). Therefore, the value of 0.496 µSv/Gycm^2^ is, in fact, the upper limit of left eye lens doses normalised to DAP for ablations.

Finally, the incorrect placement of the ZG face shield which could result in partial eye exposure might be the reason for observed differences in dose reduction presented in the quoted studies.

Regarding the effectiveness of the ZG system in reducing the doses to the chest it cannot be compared as the whole body dosimeter in this study was placed on the lead apron while in the quoted ones—under it. Therefore, no doses were measured in the referred studies and the potential non-equivalence could not be demonstrated. However, in the study of Haussen et al. (2016) one of the dosimeters was placed on the ‘outer aspect of the thyroid shield’ which allows for some comparison; 13.9 times higher doses with ceiling suspended lead shield vs. the ZG system were found by Haussen et al. (2016) on thyroid shield, while in this study 15 times higher on the left side of the chest. The observed slight difference, apart from the problematics of dynamic factors for clinical practice mentioned above, is due to different dosimeter positions (thyroid on the lead shield vs. chest on the lead apron). It also might indicate that when the ZG system is used, the radiation reaching the dosimeter placed on the thyroid passes through the lead apron rather than through the front or side lead glass.

The observed increased effectiveness of the ZG system in the thoracic area can be attributed to the doubled lead equivalence thickness of the cover in this area as compared to the ceiling shield. Moreover, the effectiveness of the ceiling shield is sensitive to its correct positioning. The last issue is particularly important for the protection of the eyes. To make the ceiling shield the most effective, the continuous adjustment of its position to the currently used projection is required (which in practice is rarely the case), while the ZG system with its side lead glass shields provides better protection even in cases of the most unfavourable projection such as LAO90 and without additional interference of the staff.

Contrary to the above conclusions, the results showed that the average doses to hands from procedures performed with ZG system were significantly higher as compared to procedures performed with ceiling suspended shield only. Indeed, the ZG system alone, in contrast to the ceiling shield, does not provide any protection for hands and forearms. The increased exposure may also be partly ascribed to the fact that operators were not experienced with ZG system which could result in different position and additional manipulations as compared to the usual practice. However, this issue was not investigated in this study.

Doses collected in the two rooms for two different procedure types performed with the ZG system provided an assessment of the exposure levels for ablation and angiography and angioplasty procedures as well as their comparison. The results show that regardless of the type of procedure the average doses were about two times higher on the left finger as compared with the right one and about ten times higher for haemodynamic procedures compared with ablations for both fingers. The average doses to the left ankle were slightly higher than average left finger doses in case of ablations but much lower (almost nine times) in case of CA and PCI procedures. Although ankles were not shielded by the ZG system the table lead curtain, which was used in both laboratories, provided the protection for lower extremities.

Regarding the doses measured with the whole body dosimeters in the electrophysiology room, they were all below the LDL, which means that given that the dose records were cumulated from several procedures, the average whole body dose per procedure for the dosimeters placed on the neck, chest and waist were 0.010 mSv or less, while in the hemodynamic room at most 0.006 mSv or 0.012 mSv for the neck and waist or chest dosimeter, respectively. However, knowing that ZG system and the ceiling suspended shield were simultaneously used in electrophysiology lab and taking into account that average values presented were assessed from the value of LDL (which numerically was the same for both procedures) and the number of procedures performed for a given measurement (which was usually lower in the case of ablations) the doses per procedure should be much lower than the evaluated upper values. The situation is different for the haemodynamic lab, where only the ZG system was used and more procedures were accumulated during one measurement, and therefore, the doses were closer to the real values.

The results of the study prove that the ZG system is characterised by outstanding effectiveness in protection of the main operator. Therefore, it could be useful in complex, high dose procedures and in particular in emergency procedures. Moreover, the advantage of such solutions such as the ZG system is that they can be simultaneously used with the ceiling shield (as it is indicated by the example of the electrophysiology lab) or with any other protection commonly used in interventional room as long as shields do not interfere with each other in practice (which might be a case in procedures, where oblique projections with large angles are used). In this study, in the case of ablation and cryoablation procedures, where both ZG system and ceiling shield were used, no such interference was observed as most of the procedures were performed in PA or oblique projections with small angles. On the other hand, this study proves that additional use of the ceiling shield would be necessary to ensure comparable protection for the hands with that provided by the standard shield. Another important advantage is that very high level of protection might be obtain without burdening the physician with heavy lead shields.

The above study has also some limitations. Due to the relatively low sensitivity of TL dosimeters they were placed during measurements without ZG system on the standard lead apron. Therefore, this study does not allow a comparison between the effectiveness of the ZG system with the standard set used in most of interventional rooms, such as the standard lead apron and the ceiling shield. Analysing the impact of the simultaneous use of these shields on the operator’s protection would be a more interesting and important issue, although more difficult to implement, which is confirmed by the results of the cited studies (Savage et al. 2013; Haussen et al. 2016).

In addition to providing superior protection, the physicians unanimously declared in the survey that the ZG system significantly increases the comfort of work compared to a standard lead apron. On the other hand, the costs of such solution is incomparably higher than of standard, normally used shields (Dabin et al. 2021). Therefore, before taking the decision on purchasing the ZG system, the hospital management should consider, on the one hand, its advantages including ergonomic properties, high level of protection from ionizing radiation and the number of personnel that would use it in practice and, on the other hand, its relatively high costs.

## Conclusions

The ZG system proved to be very effective in protecting the shielded parts of the prime operator’s body. As the ZG system does not protect hands it might be used simultaneously with the ceiling suspended lead shield to ensure protection of upper extremities but only in cases when both shielding systems do not collide with each other.

## Data Availability

The data that support the findings of this study are available from the corresponding author, upon reasonable request.
